# OSRA^QMUL^: a digital application for oral surgery risk assessment

**DOI:** 10.1038/s41405-026-00437-w

**Published:** 2026-05-01

**Authors:** Haidar Hassan, Hussein Al-Tamimi, Rawand Shado, Saffa Dean, Ravi Rathod, Paulo Oliva, Atif Matin

**Affiliations:** 1 Academic Plastic Surgery Programmes, Centre for Cutaneous Research, Blizard Institute, Faculty of Medicine and Dentistry, Queen Mary University of London, London, UK; 2 Institute of Dentistry, Royal London Dental Hospital, Barts & The London School of Medicine & Dentistry, Queen Mary University of London, London, UK; 3 School of Electronic Engineering and Computer Science, Queen Mary University of London, London, UK

**Keywords:** Dental clinical teaching, Preventive dentistry, Special care dentistry

## Abstract

**Background:**

Adherence to clinical guidelines is central to patient safety in oral surgery, yet guideline uptake in routine dental practice remains inconsistent. Traditional guideline formats, such as static PDF documents, can be difficult to navigate at the point of care. Digital decision-support tools may improve accessibility, efficiency and clinician confidence, but evidence specific to oral surgery is limited.

**Aim:**

To evaluate whether the Oral Surgery Risk Assessment (OSRA) digital application improves usability, efficiency, perceived clinical impact and user satisfaction compared with conventional PDF-based guideline access.

**Methods:**

A randomised crossover study was conducted among 30 participants from a single academic centre, including undergraduate dental students, junior dental surgeons (DFTs/DCTs) and senior clinicians. Participants used both the OSRA digital application and conventional PDF guidelines across standardised oral surgery clinical scenarios. Outcomes were assessed using a structured questionnaire covering usability, reliability and trust, efficiency and workflow, satisfaction and future use and perceived clinical impact, measured on five-point Likert scales. Paired comparisons were analysed using Wilcoxon signed-rank tests, with effect sizes reported. Qualitative free-text feedback was analysed thematically.

**Results:**

Across all domains, the OSRA application was consistently rated higher than PDF guidelines (all *p* < 0.05), with large to very large effect sizes (*r* = 0.55–0.75). The greatest differences were observed for efficiency and workflow (*r* = 0.75) and satisfaction and future use (*r* = 0.73). Participants reported reduced cognitive load, faster access to guidance and greater confidence in clinical decision making when using the app. Reliability analysis demonstrated excellent internal consistency for app-based ratings (Cronbach’s *α* = 0.96). Qualitative feedback highlighted clarity, intuitive navigation and workflow integration as key strengths, alongside requests for enhanced transparency of evidence sources and expanded functionality.

**Conclusions:**

In this pilot study, the OSRA digital application outperformed conventional PDF guidelines across all evaluated domains, suggesting that digital, point of care decision support tools can improve guideline accessibility, efficiency and user experience in oral surgery. With further development and validation, such tools have the potential to support safer, more consistent evidence-based clinical practice.

## Introduction

Patient safety in dental and oral surgery remains an established priority globally and in the UK. Research in this area is still emerging, with most studies historically conducted in hospital settings despite approximately 95% of dental care being delivered in primary care [1]. This imbalance means that safety practices in general dental practice have lagged. In fact, patient safety culture in dental and oral surgery is thought to ‘lag compared to medical specialties, especially in general dental practice’ [2]. One reason is that most dental clinics are independent businesses; around 90% of dental and oral healthcare occurs in practice settings where reporting of adverse events is often discouraged by fear of litigation or financial repercussions [2]. Such underreporting of incidents contributes to poor patient safety outcomes, as it impedes learning from errors and can lead to preventable harm. This underlines the need for better safety strategies and a stronger safety culture.

Adherence to clinical practice guidelines (CPGs) is central to safe, consistent care in dental and oral surgery. Evidence-based guidelines refine best practices to minimise risk, yet, in practice the uptake of these guidelines is often suboptimal. Evidence from the UK, USA and Europe consistently demonstrates that adherence to evidence-based dental clinical guidelines remains suboptimal across a wide range of procedures. Audits and observational studies show low baseline compliance with antibiotic prescribing guidance, with UK hospital and primary care audits reporting adherence rates of around 20–30% and US data indicating that over 80% of dental antibiotic prophylaxis prescriptions are unnecessary according to guidelines [3–5]. Similar underuse is observed beyond antibiotics: preventive guidelines such as pit-and-fissure sealant recommendations are poorly implemented, with adoption rates among general dentists ranging from below 5% to approximately 38%, despite strong supporting evidence [6, 7]. In operative dentistry, guideline-mandated practices such as rubber dam use during endodontic treatment are inconsistently followed, with surveys reporting routine use by only around 20–30% of general dentists compared with much higher uptake among specialists [8]. Infection control and safety guidance shows similar gaps, including inconsistent hand-hygiene practices and incomplete compliance with recommendations for sterile irrigants during surgical procedures, even in high-income healthcare systems [9]. Collectively, these findings highlight persistent evidence to practice gap in dentistry and oral surgery, where well-established guidelines intended to minimise risk and optimise patient outcomes are frequently underused in routine clinical care, despite evidence that targeted audit, feedback, and educational interventions can substantially improve compliance.

Key barriers to guideline adherence include lack of awareness or familiarity with the recommendations, insufficient training or confidence in applying them, time constraints during busy clinics and the inertia of ‘usual practice’ [10]. Complex or lengthy guidelines can overwhelm clinicians, especially under time pressure, making it harder to follow every step. If practitioners do not fully agree with a guideline or find it impractical in their setting, they may consciously or unconsciously diverge from it [10]. These challenges result in a sizeable gap between best-practice protocols and actual practice in dentistry and oral surgery.

Many mobile and web-based applications have been developed to support adherence to evidence-based dental clinical guidelines by translating complex recommendations into practical, point-of-care decision tools. In the UK, the Scottish Dental Clinical Effectiveness Programme (SDCEP) Dental Companion provides a mobile-friendly web platform that consolidates multiple national guidelines (e.g. periodontal disease, anticoagulant management, caries prevention) into interactive flowcharts and summaries to support real-time clinical decision-making [11]. while the SDCEP Dental Prescribing resource similarly operationalises national prescribing guidance to promote safer, guideline-concordant drug use [12]. In endodontics, the British Endodontic Society (BES) Case Assessment Tool (often referred to as the BES EndoApp) offers a structured, guideline-based scoring system to assess endodontic case complexity and guide appropriate treatment planning or referral, directly addressing known variability in guideline uptake [13]. for the BES Endodontic Case Assessment Tool/EndoApp. In the US, commercial tools such as DentalRx: Treatment Guidelines provide mobile access to treatment protocols and prescribing guidance aligned with professional recommendations, assisting clinicians in making chairside evidence-based decisions [14]. These apps demonstrate an implementation-focused approach to improving guideline adherence in dentistry by embedding evidence-based recommendations into accessible, clinician-friendly digital tools.

The Oral Surgery Risk Assessment (OSRA) digital application was conceptualised and developed through close collaboration between academic clinicians and engineers at Blizard Institute, the Oral Surgery Department and the School of Electronic Engineering and Computer Science department at Queen Mary University of London (QMUL). The objective was to create a cross-platform tool, compatible with iOS and Android systems, that would provide immediate access to oral surgery clinical guidelines and decision-support features within a single user-friendly interface.

This study investigates whether the Oral Surgery Risk Assessment (OSRA) digital application improves perceived usability, workflow efficiency and user satisfaction compared with conventional PDF-based guideline access during simulated oral surgery decision-making scenarios.

## Materials and methods

### OSRA digital application development

Development followed an iterative design methodology. The initial coding and interface prototypes were built by the software engineering team using a modular architecture to allow rapid feature testing and subsequent refinements. Early-stage prototypes were repeatedly trialled by a small panel of oral surgery trainees and senior clinicians, who provided structured feedback on usability, layout, and clinical relevance. Each feedback cycle informed further code revisions, user-interface adjustments, and feature optimisation to ensure the tool met both technical performance standards and the practical needs of end-users [15–17].

Functionality testing included assessment of app stability, response speed, and integration of evidence-based guideline content. Multiple beta versions were released internally for pilot testing before the final prototype was approved for use in the main study. By the time of participant recruitment, the OSRA app incorporated streamlined navigation menus, embedded clinical checklists aligned with national safety and visual outputs designed to support rapid information retrieval during clinical decision-making.

**Fig. 1:**
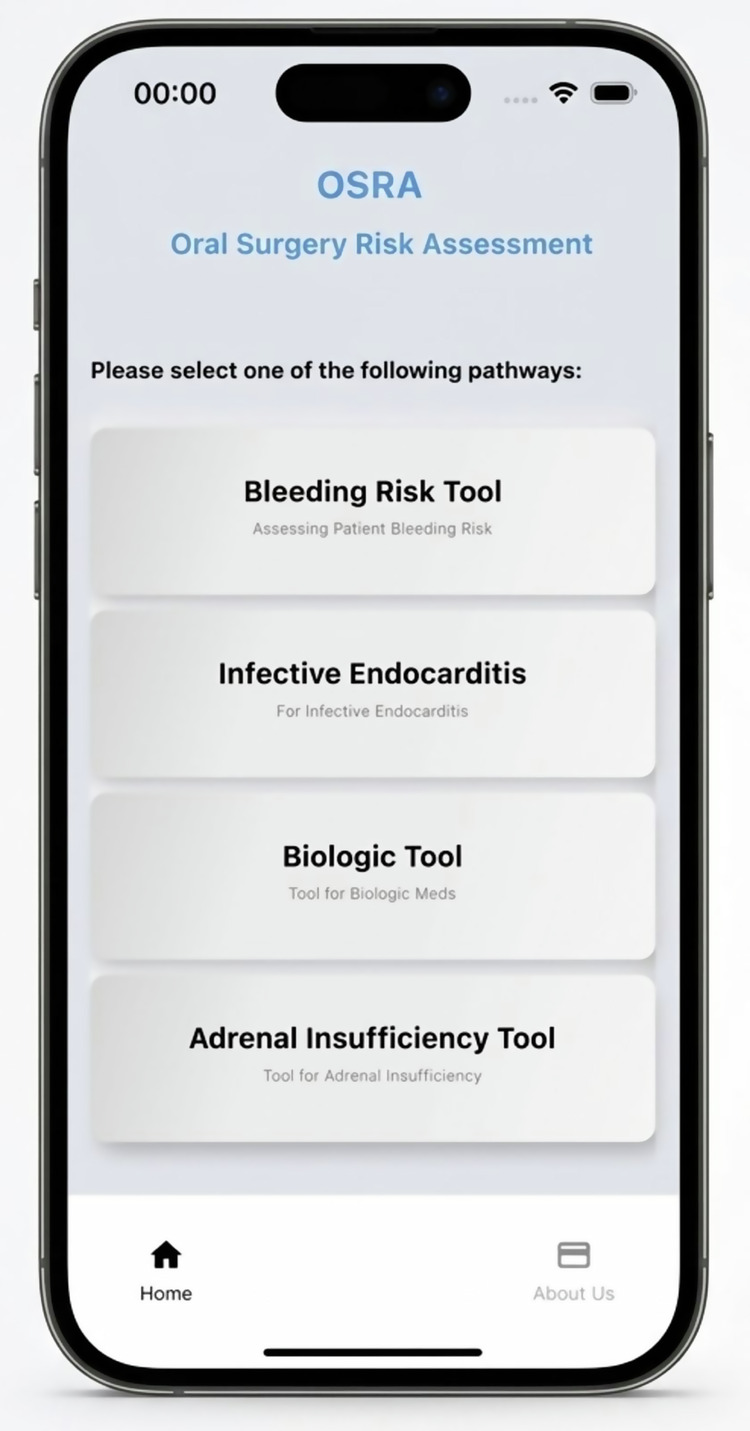
OSRA app mobile interface. OSRA digital App user interface displaying four core clinical decision-support tools: bleeding risk tool, infective endocarditis tool, biological tool, and adrenal insufficiency tool.

The OSRA application incorporates four core clinical decision-support tools, each aligned with established national guidance addressing high-risk scenarios in oral surgery (Fig. 1).

- Bleeding risk tool: an antiplatelet and anticoagulant management tool, based on Scottish Dental Clinical Effectiveness Programme (SDCEP) guidance, supports peri-operative decision-making for patients receiving antithrombotic therapy, including warfarin and direct oral anticoagulants, with structured pathways for bleeding risk assessment and treatment planning [11].
- Infective endocarditis tool: an infective endocarditis prophylaxis tool, aligned with SDCEP guidance, supports assessment of cardiac risk and appropriate antibiotic prescribing, reinforcing guideline-consistent practice and antimicrobial stewardship [18].
- Biological tool: a biologic Disease-Modifying Anti-Rheumatic Drugs (DMARD) safety tool, developed in accordance with established biologic therapy guidelines, supports management of patients receiving biologic agents by highlighting relevant peri-operative risk considerations [19].
- Adrenal insufficiency tool: a steroid management tool, informed by NICE and NHS specialist Pharmacy Service guidance, assists clinicians in identifying patients at risk of adrenal suppression and determining the need for peri-operative steroid supplementation for dental and minor oral surgery procedures [20].

### Study design and setting

The study adopted a randomised crossover design to ensure that each participant experienced both interventions: the OSRA digital application and the conventional PDF guideline format. Ethical approval was obtained from the Queen Mary University of London Research Ethics Committee (reference: QME24.0621). Participants were recruited from Queen Mary University of London (QMUL) and Barts Health NHS Trust across multiple training levels, including final-year dental students, Dental Foundation Trainees (DFTs), Dental Core Trainees (DCTs), and senior clinicians actively involved in oral surgery or related clinical specialties such as maxillofacial surgery, restorative dentistry, and special care dentistry. Recruitment took place between January and June 2025 via institutional mailing lists, departmental briefings, and professional networks. Information sheets describing the study objectives, procedures, and voluntary nature of participation were distributed electronically. Inclusion criteria required participants to be engaged in clinical practice or training within the field of dental and oral surgery, and able to provide informed consent. Exclusion criteria were refusal to participate. Participation was entirely voluntary, with the option to withdraw at any point without providing a reason. All questionnaire responses were collected anonymously, and no personally identifiable information was recorded. Data were stored on password-protected institutional systems accessible only to members of the research team.

The study aimed to recruit 30 participants, divided equally into three cohorts: undergraduate dental students (*n* = 10), postgraduate dentists including DFTs and DCTs (*n* = 10), and senior clinicians (*n* = 10). Written informed consent was obtained from all participants before study commencement.

**Fig. 2:**
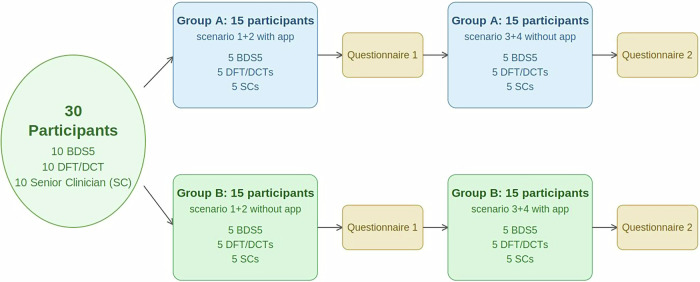
Study crossover design and allocation of interventions.

Participants were randomised into two equal groups (Group A and Group B) using a computer-generated random number sequence to ensure allocation concealment. Group A used the OSRA application for scenarios 1 and 2, while Group B used conventional PDF guidelines for these cases. For scenarios 3 and 4, the conditions were reversed: Group A switched to conventional guidelines and Group B used the OSRA app. This crossover design ensured that every participant experienced both interventions while controlling for order effects (Fig. 2).

#### Standardised clinical scenarios

Four predefined simulated clinical scenarios were developed for the study, each corresponding to one of the OSRA application’s four core decision-support areas: anticoagulant and antiplatelet management, infective endocarditis prophylaxis, biologic therapy risk assessment, and adrenal insufficiency/steroid supplementation. Each scenario was written in a standardised format and presented equivalent core clinical information, including relevant medical history, medication history and the planned oral surgery procedure. The scenarios were reviewed by the study team and senior oral surgery clinicians prior to study commencement to ensure clinical realism, consistency, and comparability across cases (Supplement 1).

#### Sample size considerations

This study was designed as a pilot exploratory evaluation of the OSRA application. As no prior studies evaluating oral surgery decision-support tools in this context were available, there were no data to perform a formal a priori power calculation. Instead, the sample size was chosen pragmatically to allow preliminary estimation of effect sizes and response variability across the evaluated domains. These estimates may inform the design and power calculations of future confirmatory studies.

#### Questionnaire design

The questionnaire was designed following a comprehensive review of existing healthcare technology evaluation instruments, including the DeLone and McLean (2003) Information Systems Success Model. The questionnaire items covered five core domains: usability, reliability and trust, efficiency and workflow, satisfaction and future use and clinical impact using a five-point Likert scale (1 = strongly disagree, 5 = strongly agree).

### Data collection and analysis

All statistical analyses were conducted using Python. Responses were collected using five-point Likert scales (Strongly Disagree to Strongly Agree) and treated as ordinal data. For descriptive analysis, responses were grouped thematically and summarised using clustered diverging stacked bar charts, displaying the percentage distribution of responses across Likert categories for each format.

For inferential analysis, questionnaire items were grouped into five predefined domains: usability, reliability and trust, efficiency and workflow, satisfaction and future use, and clinical impact. For each participant, a domain-level score was calculated separately for the App and PDF conditions as the mean Likert rating across all items within that domain. This composite scoring approach was used to summarise responses across conceptually related items at participant level before paired comparison between formats.

Paired comparisons between the App and PDF formats were performed using Wilcoxon signed-rank tests, reflecting the ordinal nature of the response data and the within-participant crossover study design. Effect sizes were calculated as r, derived from the standardised Wilcoxon test statistic and interpreted using conventional thresholds of 0.1, 0.3, and 0.5 for small, moderate, and large effects, respectively. To account for multiple comparisons across the five domain-level tests, a Bonferroni correction was applied. All tests were two-tailed, with statistical significance set at *p* < 0.05.

### Primary and secondary outcomes

The main outcome domain in this study was efficiency and workflow, selected because the OSRA application was designed primarily as a point-of-care decision-support tool to facilitate rapid access to relevant oral surgery guidance. Secondary outcome domains were usability, reliability and trust, satisfaction and future use and perceived clinical impact.

### Ethical approval

Ethical approval for this study was obtained from the Queen Mary University of London Research Ethics Committee (Reference: QME24.0621).

## Results

### Overall comparison between app and PDF formats

**Fig. 3:**
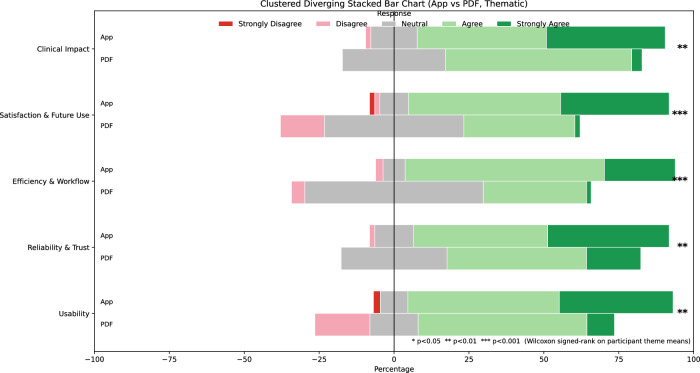
Clustered diverging stacked bar chart comparing participant responses to the App and PDF formats across five thematic domains: usability; reliability and trust; efficiency and workflow; satisfaction and future use; and clinical impact. Responses were assessed using a five point Likert scale ranging from Strongly Disagree to Strongly Agree, with negative values indicating levels of disagreement and positive values indicating levels of agreement.

The primary outcome domain, efficiency and workflow, showed the largest difference between formats (Table 1). Across all domains, the App demonstrated a consistent rightward shift in response distributions compared with the PDF. The App was associated with a higher proportion of Agree and Strongly Agree responses and fewer Neutral and Disagree responses, whereas the PDF showed a greater concentration of neutral and lower-agreement responses. These visual trends were consistent across all assessed domains (Fig. 3).

**Table 1:** Summary of domain-level comparisons between the OSRA application and PDF guideline formats.

Domain	*p*-value	Effect size (*r*)
Usability	0.001	0.63
Reliability and trust	0.007	0.55
Efficiency and workflow	<0.001	0.75
Satisfaction and future use	<0.001	0.73
Clinical impact	0.006	0.65

Paired Wilcoxon signed-rank test results comparing participant ratings of the OSRA application and conventional PDF guideline format across five questionnaire domains. Effect sizes (*r*) are derived from the standardised Wilcoxon statistic. Bonferroni correction was applied for the five comparisons.

Paired comparisons between the App and PDF were conducted using Wilcoxon signed-rank tests, reflecting the ordinal nature of Likert-scale data and the within-participant crossover design. Analyses were performed on participant-level theme scores, calculated as the mean Likert rating across all items within each domain. Effect sizes were quantified using r, with values of 0.1, 0.3, and 0.5 conventionally interpreted as small, moderate, and large effects, respectively. All reported domain-level differences remained statistically significant after Bonferroni correction for the five prespecified comparisons.

### Usability

Usability ratings were higher for the App compared with the PDF (*p* = 0.001), with alarge effect size (*r* = 0.63). The median paired difference indicated that App ratings exceeded PDF ratings by approximately two-thirds of a Likert scale point.

Qualitative feedback strongly supported these quantitative findings. Participants frequently described the App as clearer, more intuitive, and easier to navigate than static PDF guidelines. However, suggestions for further usability enhancement were consistently raised, including improvements in visual design, clearer block text, and a more intuitive layout. Participants across all experience levels emphasised the value of a short onboarding guide to support first-time users and clarify navigation pathways within the App.

### Reliability and trust

Reliability and trust scores were also higher for the App than for the PDF (*p* = 0.007), again with alarge effect size (*r* = 0.55). Participants reported greater confidence in the App’s outputs and decision-support functionality.

Despite this overall confidence, qualitative feedback revealed a consistent desire for greater transparency regarding the evidence base underpinning App recommendations. Participants requested clearer signposting of source guidelines, direct links to original documents, explicit citation of evidence, and greater clarity around how recommendations were derived. This was particularly emphasised for complex clinical areas such as anticoagulation management, infective endocarditis prophylaxis, and biologic therapies.

### Efficiency and workflow

The largest quantitative differences were observed in the Efficiency and Workflow domain. App ratings were substantially higher than PDF ratings (*p* < 0.001), with a very large effect size (*r* = 0.75). Median paired differences approached one full Likert scale point, indicating a clinically meaningful perceived improvement in efficiency when using the App.

This finding was strongly reinforced by qualitative feedback, particularly among senior clinicians and dental core trainees. Participants reported that accessing guidance through the App required less effort and was perceived to streamline workflow compared with navigating lengthy PDF documents. Nevertheless, workflow-related limitations were also identified. These included difficulties selecting multiple conditions or medications, the need to reset the App to avoid incorrect outputs, and challenges in managing patients with multiple comorbidities within a single workflow.

### Satisfaction and future use

Satisfaction and future use ratings demonstrated a clear advantage for the App over the PDF (*p* < 0.001), with a very large effect size (*r* = 0.73). Participants were more likely to report satisfaction with the App and to indicate an intention to continue using it in future clinical practice.

Qualitative feedback highlighted positive perceptions of the App’s clarity, drop-down menus, and overall design. Suggestions for improvement focused on enhanced aesthetics, more consistent visual cues, and the inclusion of summary outputs when multiple conditions or medications are present. Participants also proposed the integration of virtual patient notes, allowing medication lists and comorbidities to be entered once and retained across interactions.

### Clinical impact

Clinical impact ratings were higher for the App compared with the PDF (*p* = 0.006), with alarge effect size (*r* = 0.65). This indicates that participants perceived the App as having greater potential to support clinical decision-making during oral surgery risk assessment scenarios.

Qualitative feedback identified several priorities for further development, including expansion of the toolset, more tailored recommendations for specific cardiac conditions (e.g. prosthetic versus biological valve replacements), clearer guidance on antibiotic dosing rather than general prophylaxis advice, and incorporation of additional patient-specific variables such as renal function.

### Reliability analysis

Internal consistency analysis revealed excellent reliability for the OSRA app questionnaire (Cronbach’s *α* = 0.9615; McDonald’s *ω* = 0.9658) compared to the PDF version (*α* = 0.8826; *ω* = 0.9085). These results indicate that the questionnaire items measured coherent constructs with minimal measurement error and that participants rated the OSRA app in a highly consistent manner.

## Discussion

In this pilot study, the OSRA app outperformed conventional PDF guidelines across all evaluated domains, indicating a clear user preference for the interactive, app-based format. Participants rated the app higher on usability, trust, efficiency, satisfaction and perceived clinical impact, with differences on the order of two-thirds to one full Likert scale point favouring the app. These gains were practically meaningful, as reflected by the large effect sizes (*r* = 0.55–0.75) observed. Such uniformly positive results suggest that embedding evidence-based oral surgery guidance into a well-designed digital application can substantially enhance clinicians’ perceived experience and confidence when accessing guideline information relative to traditional static documents. Notably, this crossover trial design controlled for ordering effects and exposed every participant to both formats, adding weight to the conclusion that the app’s advantages were consistent and not merely due to individual or cohort biases. Moreover, the high internal consistency of responses (Cronbach’s *α* = 0.96 for app-based ratings, versus 0.88 for PDF) reinforces that participants had a coherent and reliable reaction favouring the app; a Cronbach’s *α* above 0.9 is generally considered excellent in terms of reliability [21]. In practical terms, users across all experience levels found the OSRA app easier to navigate and more helpful for clinical scenarios than the paper PDF, signalling a broadly applicable benefit.

Our findings align closely with the growing body of literature on mobile guideline and decision-support tools in healthcare. For example, Hakes et al. [22]. reported high usability and satisfaction when trauma providers transitioned from paper to a mobile guideline app: 88.9% of surveyed users were satisfied with the app and willing to recommend it, and 63% agreed it improved their clinical care for trauma. This mirrors our participants’ strong endorsement of the OSRA app’s usefulness and their belief that it could positively impact patient management. In another study, Bottini and colleagues [23]. found that introducing a web-based decision support system for dental students significantly changed their clinical management choices compared to not using the tool (*p* < 0.05). This suggests that beyond just perception, digital tools such as OSRA may support clinicians in accessing guideline-based recommendations more efficiently. Our participants likewise felt the app would have greater clinical impact than the PDF, an expectation supported by such findings in the literature.

The enhancement in efficiency and workflow we observed is also well documented in prior studies. Mobile tools often reduce the time and cognitive load required to retrieve information. Kamath et al. [24]. For instance, showed that general dentists took nearly four times longer to write paediatric prescriptions by hand (mean; 199 s) than to complete them with an app (52 s), with the app yielding far higher accuracy and user satisfaction. While our study did not directly measure task times, the higher Efficiency domain scores (with a very large effect size, *r* = 0.75) indicate that clinicians felt the app streamlined their workflow noticeably; a sentiment echoed qualitatively by senior clinicians and trainees who reported saving time and effort. This accords with known principles for effective clinical decision support, notably that ‘speed is everything’ and tools must fit into the user’s workflow to be truly useful [25]. By delivering guideline advice in an on-demand, interactive format, the OSRA app appears to satisfy these criteria better than a static PDF; and hence potentially reducing the effort required to access relevant guidance during clinical decision-making.

Participants overwhelmingly favoured the app’s usability and expressed greater satisfaction and intention for future use compared to the PDF. This is consistent with other evaluations of clinical apps. In trauma care, 92.6% of providers considered a guideline app to be comprehensive and well organised and nearly 89% were satisfied overall [22]. Similarly, in a dental context, over 95% of dentists in a recent study were satisfied or very satisfied with using an app for paediatric drug prescribing [24]. Together with our results, these reports suggest that when a mobile application is thoughtfully designed and targets a genuine clinical need, user acceptance can be extremely high across different healthcare domains. It is worth noting that our participant pool included undergraduate students, recent graduates and senior clinicians; yet the app’s benefits were noted across all these groups. This broad appeal is encouraging for adoption; it suggests even experienced clinicians (who may be accustomed to traditional guidelines) can see value in the app format when the improvements in clarity and convenience are evident. The qualitative feedback in our study indicated that participants found the app ‘clearer and more accessible’ than the PDF, reinforcing the idea that user-centric design can overcome initial resistance and improve guideline uptake. These findings resonate with the Technology Acceptance Model framework, wherein perceived ease of use and usefulness drive intentions to use a new technology. In our case, the app clearly scored higher on both ease and usefulness, likely underpinning the greater stated intention for continued use compared to the PDF.

One notable theme was that despite higher trust scores for the app, participants desired more transparency about the underlying guidelines and evidence. Many requested features such as direct links to source guideline documents, citations for recommendations and clarity on how the advice is derived; especially for complex areas such as anticoagulation, endocarditis prophylaxis or biologic therapies. This feedback highlights that trust in clinical decision apps is partly predicated on seeing the evidence behind the curtain. The wider literature strongly supports this view: recently published guidelines for health apps (e.g. the Xcertia mHealth App Guidelines) emphasise that apps must clearly communicate their content sources, ideally citing credible guidelines or studies and make this information easy for the user to find. Unfortunately, many apps bury or omit their evidence sources, which can erode user confidence [26]. In our study, the OSRA app was built on recognised guidelines (as noted in the ‘Methods’), which likely gave it baseline credibility; no participant expressed fundamental doubts about the *accuracy* or safety of the app’s guidance. However, providing in-app citations and hyperlinks to the original guideline texts would likely further strengthen users’ trust and willingness to rely on the app in practice. This is a clear area for improvement. Ensuring transparency of recommendations; for example, by displaying a ‘source’ or ‘info’ button next to each recommendation that reveals the guideline reference; could address users’ calls for greater reassurance that the app’s advice is evidence-based. By doing so, OSRA would adhere to best practices for building and maintaining trust in decision support tools, where accurate and up-to-date evidence documentation is essential.

### Study strengths and limitations

The present study is among the first to rigorously compare an oral surgery guideline mobile app against traditional PDF guidelines using a randomised crossover design. This within-subject approach strengthens the validity of our findings by minimising inter-participant variability and learning effects. All participants were given equal exposure to both formats and the order of exposure was counter-balanced, which enhances confidence that the observed differences truly stem from the intervention (app vs PDF) rather than extraneous factors. We also employed both quantitative Likert-scale evaluations and qualitative feedback, allowing us to triangulate the data; the statistical results were supported and enriched by participants’ own explanations and comments. The consistency between the quantitative trends (all favouring the app) and the qualitative remarks (which largely praised the app while suggesting improvements) is a notable strength, as it indicates a coherent narrative of the app’s benefits and areas for enhancement.

Despite these strengths, there are important limitations to consider. First, the sample size was modest (*n* = 30), drawn from a single institution and divided equally among students, junior dentists and senior clinicians. While this provided a diverse range of experience, the overall number of participants is small, which may limit the generalisability of the findings. That said, our sample size is comparable to other exploratory studies in this domain (for example, similar app usability studies often include 20–30 participants) and was sufficient to detect large effect sizes across all domains [24]. A related point is potential selection bias: those who volunteered for the study may have been more tech-savvy or enthusiastic about digital tools than the average clinician. If so, the app’s reception in a more reluctant population might be less enthusiastic.

The exploratory nature of this pilot study and the relatively small sample size mean that the findings should be interpreted cautiously. However, the results provide preliminary effect-size estimates that may inform the design and sample size calculations of future multi-centre larger-scale studies evaluating decision-support tools in dental education and clinical practice.

In addition, there is a risk of courtesy bias, as a proportion of participants were recruited from the same institution in which the application was developed and included students, trainees, and colleagues of the investigators. Participants may therefore have provided more favourable ratings due to perceived social desirability, professional familiarity, or a reluctance to express criticism, particularly in a supervised academic environment. While the anonymous questionnaire format may have mitigated this effect to some extent, courtesy bias cannot be excluded and should be considered when interpreting the uniformly positive evaluations of the app.

Our study adds to the evidence that digital platforms can enhance the delivery and utilisation of clinical guidelines in dentistry and oral surgery. By demonstrating robust user preference for the OSRA app, we highlight an opportunity for dental education and practice to leverage mobile technology for guideline dissemination. In practical terms, an app like OSRA could serve as a point-of-care decision support tool, helping clinicians quickly find tailored guidance for managing patients on anticoagulants, at risk of endocarditis, on specialty medications, and so forth. The high satisfaction and reported intention to continue using the app suggest that if made available, such a tool might see good uptake among its target users. This may facilitate easier access to evidence-based protocols at the point of care, especially in areas where guidelines exist but practitioners find them cumbersome to consult in the middle of a clinic (e.g. recalling prophylaxis recommendations or drug management protocols from a dense PDF). Additionally, the app format allows for real-time updates in a way static PDFs cannot. Participants did not explicitly mention this in feedback, but other authors have noted that mobile guidelines can be updated centrally to reflect the latest evidence or policy changes [22]. For example, if new antibiotic prophylaxis guidelines are released, the app could push an update so that all users immediately have the current recommendations; an important advantage given that several participants valued the app’s reliability and presumably expect it to stay up to date.

As this study used simulated scenarios and participant perceptions rather than objective clinical performance measures, the findings should not be interpreted as direct evidence of improved clinical outcomes or patient safety.

## Conclusion

In summary, this study demonstrates that an evidence-based oral surgery risk assessment digital app can improve clinicians’ experience in accessing and applying guidelines, compared to conventional PDF documents. The OSRA app was associated with higher confidence, and better user satisfaction, which are all important for the successful uptake of clinical guidelines in practice. These findings are in line with wider literature showing the benefits of mobile guideline delivery in healthcare. With improvements and integration, digital applications like OSRA can help ensure that the best clinical guidelines truly inform everyday patient care, thereby supporting the integration of digital guideline tools in dental education and clinical practice.

## Supplementary information

Study Scenarios

## Data Availability

The datasets generated and/or analysed during the current study are available from the corresponding author upon reasonable request.
