# Supplementary material for: OSRAQMUL: a digital application for oral surgery risk assessment
Source: BDJ Open. 2026 May 1;12:43. doi: 10.1038/s41405-026-00437-w (PMC13133352; doi:10.1038/s41405-026-00437-w)

Clinical Cases

OSRA APP

Presenting Complaint:
Site: UR7
Onset: 18 months ago
Character: Pulsatile pain during episodes, followed by throbbing.
Radiates: Pain irradiates to the head.
Associated symptoms; Episodes managed twice with antibiotics.
Time/duration: Previously constant pain, only improving after antibiotics.
Exacerbating/relieving: Biting down in the affected area.
Severity: 6-7/10

Medical History :
Myocardial infarction 5 years ago, treated with stent placement.

Medications:
Amlodipine 5mg OD
Rivoroxaban 15g OD - lifelong

Allergies:
NKDA

Social History:
Smoker 5x/day
<14 units alcohol/week

Extra-Oral:
Bilateral symmetry observed; no significant findings.

Intra-Oral:
Soft Tissues: No abnormalities detected.

Hard Tissues:
UR7: Broken down, tender to percussion, grade 1 mobile, significant pocketing.

Diagnosis:

UR7 – Chronic apical periodontitis

Planned Treatment

XLA UR7

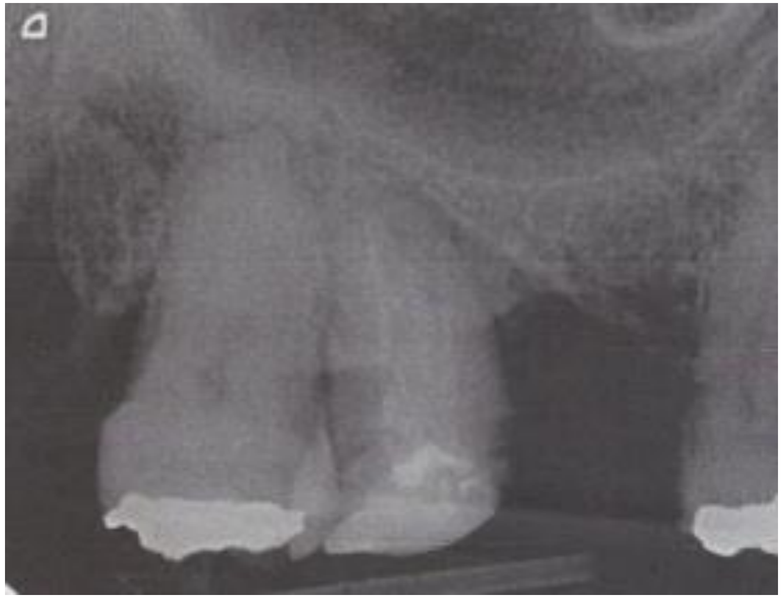

Presenting Complaint:
Site: UR6
Onset: 6 months ago
Character: Constant throbbing
Radiates: Pain irradiates to the head and ear.
Associated symptoms; Swelling managed with antibiotics.
Time/duration: Constant
Exacerbating/relieving: Biting down in the affected area. Slight pain relief with painkillers
Severity: 9/10

Medical History :
Kindey transplant
Gout

Medications:
Prednisolone 6mg OD
Allopurinol 100mg OD

Allergies:
Ibuprofen

Social History:
Non smoker
Nil alcohol

Extra-Oral:
Tender to palpate over right cheek
No other significant findings.

Intra-Oral:
Soft Tissues: Draining buccal sinus adjacent to UR6

Hard Tissues:
UR6: Gross caries, tender to percussion and palpation. Suppuration

Diagnosis:
UR6 – Chronic apical periodontitis

Planned Treatment
XLA UR6

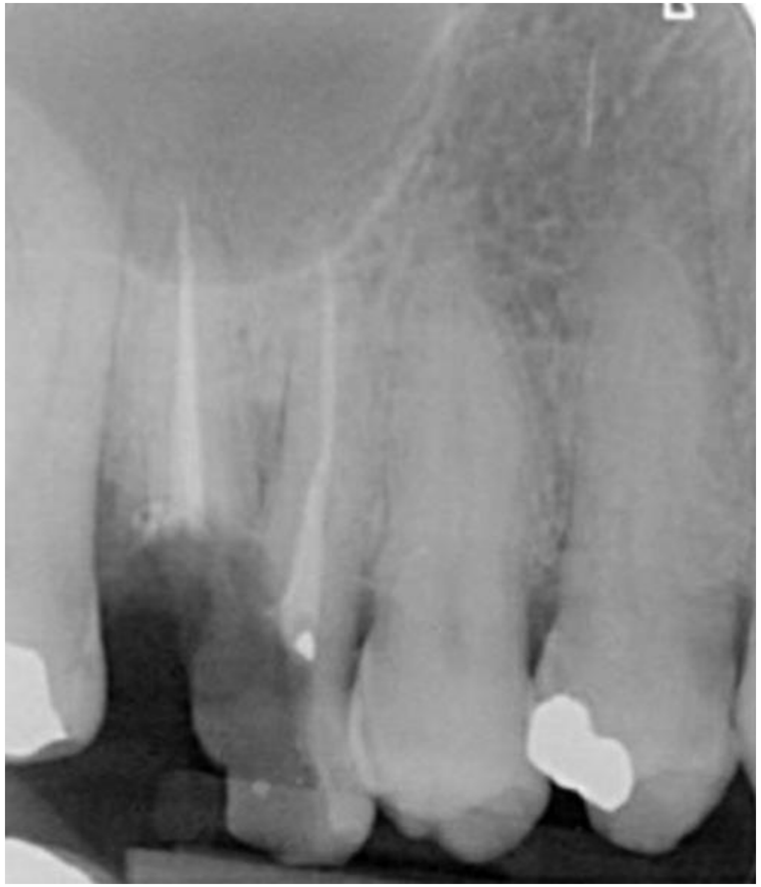

Presenting Complaint:
Site: UR4
Onset: 2 days ago
Character: Constant aching pain
Radiates: Nil
Associated symptoms; Wobbly
Time/duration: Constant
Exacerbating/relieving: Sharp pain on eating
Severity: 10/10

Medical History :
Crohn's Disease
Hypertension
Gastroesophageal Reflux Disease

Medications:
Ramipril
Omeprazole
Infliximab infusions every 8 weeks

Allergies:
Penicillin

Social History:
Ex-smoker, quit 2 years ago
Current vaper
<14 units alcohol/week

Extra-Oral:
Bilateral symmetry observed; no significant findings.

Intra-Oral:
Soft Tissues:
Gingival bruising around UR4
Hard Tissues:
UR4: grade 2 mobile, tender to percussion, tender to palpation

Diagnosis:
UR4 – apical root fracture

Planned Treatment
XLA UR4

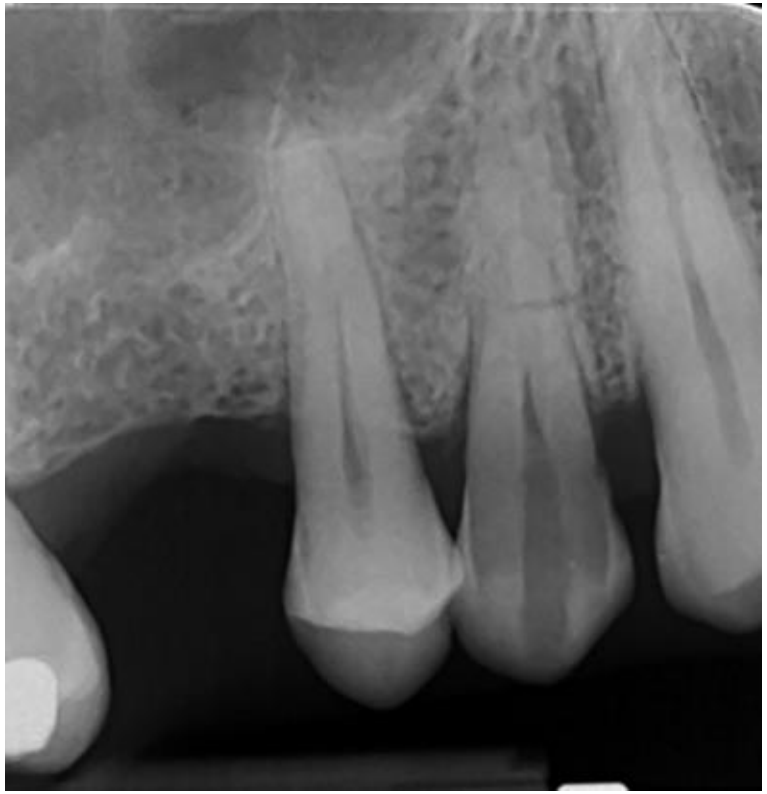

Presenting Complaint:
Site: LR8
Onset: 2 months ago
Character: Sharp, lingering pain for 1-2
Radiates: Nil
Associated symptoms; Nil
Time/duration: 10-15 minutes
Exacerbating/relieving: Sweet foods makes it worse. Pain managed with painkillers
Severity: 7/10 at its worse

Medical History :
Mitral valve replacement.
Previous infective endocarditis.

Medications:
Bisoprolol 2.5mg OD
Aspirin 75mg OD

Allergies:
Plasters

Social History:
Non smoker
Nil alcohol

Extra-Oral:
Bilateral symmetry observed; no significant findings.

Intra-Oral:
Soft Tissues: No abnormalities detected.

Hard Tissues:
LR8: Mesially mpacted with buccal caries, not tender to percussion. Hypersensitive to endofrost, lingering response.

Diagnosis:

LR8 - mesially impacted, buccal caries, reversible pulpitis

Planned Treatment

XLA LR8

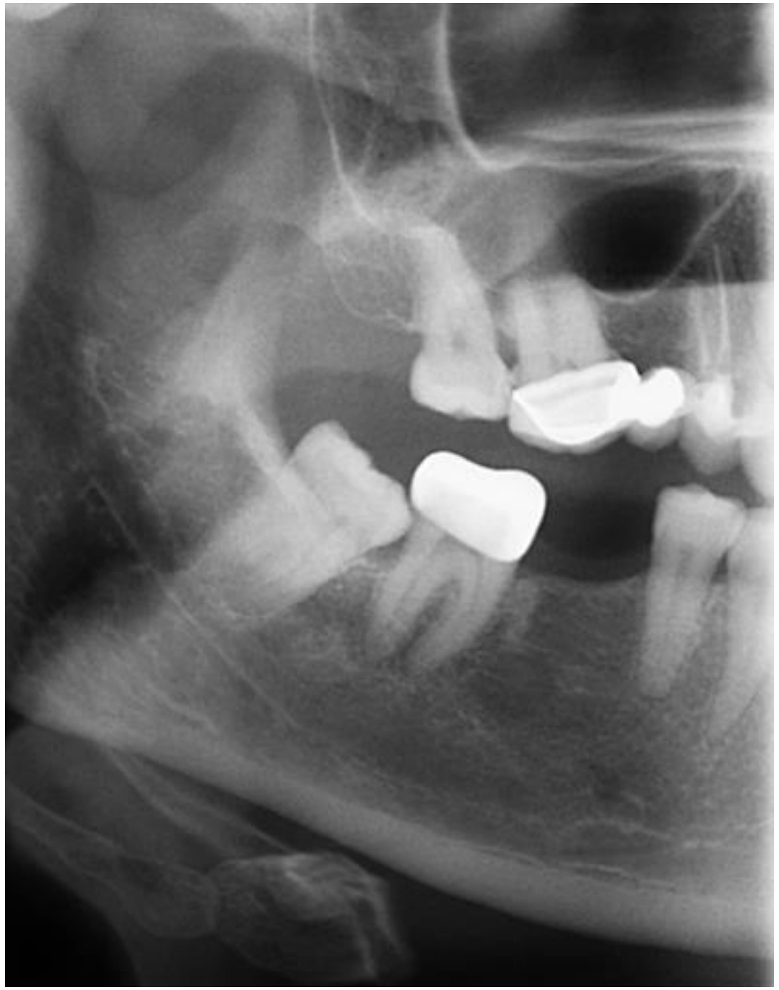

Supplement: Supplementary file 1 — Study Scenarios [file 41405_2026_437_MOESM1_ESM.pdf]
